# Shear-activation of mechanochemical reactions through molecular deformation

**DOI:** 10.1038/s41598-024-53254-2

**Published:** 2024-02-05

**Authors:** Fakhrul H. Bhuiyan, Yu-Sheng Li, Seong H. Kim, Ashlie Martini

**Affiliations:** 1 Department of Mechanical Engineering, University of California Merced, 5200 N. Lake Road, Merced, CA 95343 USA; 2 Department of Chemical Engineering and Materials Research Institute, Pennsylvania State University, University Park, PA 16802 USA

**Keywords:** Computational chemistry, Molecular dynamics, Reaction mechanisms, Physical chemistry, Reaction kinetics and dynamics

## Abstract

Mechanical stress can directly activate chemical reactions by reducing the reaction energy barrier. A possible mechanism of such mechanochemical activation is structural deformation of the reactant species. However, the effect of deformation on the reaction energetics is unclear, especially, for shear stress-driven reactions. Here, we investigated shear stress-driven oligomerization reactions of cyclohexene on silica using a combination of reactive molecular dynamics simulations and ball-on-flat tribometer experiments. Both simulations and experiments captured an exponential increase in reaction yield with shear stress. Elemental analysis of ball-on-flat reaction products revealed the presence of oxygen in the polymers, a trend corroborated by the simulations, highlighting the critical role of surface oxygen atoms in oligomerization reactions. Structural analysis of the reacting molecules in simulations indicated the reactants were deformed just before a reaction occurred. Quantitative evidence of shear-induced deformation was established by comparing bond lengths in cyclohexene molecules in equilibrium and prior to reactions. Nudged elastic band calculations showed that the deformation had a small effect on the transition state energy but notably increased the reactant state energy, ultimately leading to a reduction in the energy barrier. Finally, a quantitative relationship was developed between molecular deformation and energy barrier reduction by mechanical stress.

## Introduction

Mechanochemistry is the study of chemical reactions activated by mechanical force exerted on chemical species through processes such as grinding, milling, or shearing. Despite being an age-old concept, mechanochemical reactions have received less attention than other conventional reaction processes driven by heat, photons, or electrons. Recently, however, mechanochemistry has paved the way for many novel manufacturing, synthesis, and engineering processes that eliminate the need for a solvent, and thus, can be more energy efficient and safer than conventional technologies^1–5^. Other engineering applications where mechanochemistry plays a crucial role include surface functionalization^6^, chemical mechanical polishing^7^, polymer processing^2,8^, and tribology^9,10^. Depending on the specific application, chemical reactions can be mechanochemically activated without heat by applying normal stress, shear stress, or a combination of both normal and shear stress.

Normal stress can be either compressive or tensile and applied to reactant molecules through methods including atomic force microscopy (AFM), optical tweezers, or ultrasonication^11,12^. Compressive stress has been shown to drive the mechanochemical decomposition of alkyl thiolate based mechanophores^13,14^, or to accelerate Diels–Alder reactions^15,16^, while tensile stress has been widely used to study the mechanochemical reactions in mechanophore-embedded polymers^17,18^, surface functionalization with single molecules^6^, and the unfolding mechanisms of proteins^19–21^. On the other hand, shear-driven mechanochemical reactions are usually carried out through grinding, milling, or rubbing motion^3,22–24^ that transfers the mechanical energy of surfaces in relative motion to the reactant molecules. Shear-driven reactions also dominate the chemistry at sliding interfaces in tribological systems^9,10,25–27^.

The reaction kinetics of a mechanochemical reaction can be modeled using a stress-assisted thermal activation model, also commonly known as the Bell model^28^. According to the Bell model, the reaction rate constant of a mechanochemical reaction, *k*, can be described using a modified Arrhenius-like equation as,

1 $$k=A {\text{exp}}\left(-\frac{{E}_{a}-{E}_{m}}{{k}_{B}T}\right)$$

where *A* is the pre-exponential factor, *E*_*a*_ is the thermal activation energy, *E*_*m*_ is the mechanical energy which is a function of shear stress $\tau$ for shear-driven reactions as expressed by Equation (S2), *k*_*B*_ is the Boltzmann constant, and *T* is the temperature. The Bell model quantifies the concept of increasing the rate constant of a reaction by providing mechanical energy which effectively lowers the overall activation energy. However, the mechanical energy term, *E*_*m*_, is not fully understood. This is, in part, because its magnitude is coupled with the magnitude of *E*_*a*_, e.g., a reaction with higher thermal activation energy will require more mechanical energy to proceed. Also, mechanochemical reactions can follow completely different reaction pathways than their thermally driven counterparts^20,29^ rendering the relation between *E*_*a*_ and *E*_*m*_ more complex. Importantly, the Bell model provides no information about the mechanism through which mechanical energy can activate a reaction^30,31^.

It has been proposed that the mechanism underlying mechanical activation is deformation of reactants from their thermally and chemically stable conformation^30,32^. Macroscale experiments have shown a connection between mechanochemical activation and physical deformation of material by measuring applied strain. For example, the mechanochemical response of a dimeric anthracene-based mechanophore was investigated by compressing a mechanophore-embedded composite, revealing that the mechanophores exhibited activation upon deformation beyond a critical strain threshold^33^. Similar observations were made for the mechanochromic response of spiropyran-linked elastomers under compression and tension tests, demonstrating that a threshold strain was essential to initiate the mechanochemical reaction^34^. Such critical or threshold strain observed in macroscale experiments suggested that mechanochemical activation was triggered by physical deformation of mechanophores. Stress-induced deformation has been investigated at the molecular level by measuring change in molecular length under tension. AFM studies conducted with proteins and mechanochromic mechanophores showed that stretching the mechanophore molecules changed their length and molecular structure, eventually reshaping the potential energy surface of a reaction^12,20,21,35,36^. Surface functionalization with a single molecule was achieved by first attaching a polymer to the surface using AFM and then stretching the polymer chain between the tip and the surface to cause a chain dissociation, leaving the desired chemical species on the surface without the need for additional reactants or catalysts^6^. Such extension in polymer length has been shown to deform bond lengths and angles in the polymer backbone^37,38^. AFM experiments on the mechanochemical decomposition of methyl thiolate molecules revealed that the decomposition reactions could not be driven effectively below a critical stress threshold required for the reactant molecules to buckle^14^. However, directly observing molecular deformation is challenging with experiments, so they are often complemented by computational tools.

Quantum chemistry-based calculations using an extreme pressure polarizable continuum model showed molecular deformation during mechanochemical activation under hydrostatic stress by calculating the change in the volume of the van der Waals cavity of reactant molecules^39,40^. Density functional theory (DFT) calculations showed how molecular structure could be affected by mechanical stress during pericyclic reactions. Specifically, cyclobutene and benzocyclobutene under mechanical stress were found to undergo thermally forbidden electrocyclic ring opening reactions^11,41^. Such reactions could not be explained by the typical pericyclic selection rule since the electronic structure of the reactant under mechanochemically distorted reaction pathway remained similar to the electronic structure in thermal pathway^42^. DFT calculations also revealed that the optimized structure of reactant molecule under mechanical stress deviated from the fully relaxed molecule and that mechanical stress could modify the potential energy surface by deforming C–C bonds^41,43^. DFT-calculated reaction trajectories for [4 + 2] Diels–Alder cycloadditions between anthracene and dienophiles showed that the reaction energy barrier decreased with increasing distortion of the anthracene C–C–H angle^16^. Although first-principles or DFT-based calculations provide accurate description of the reactant structure and energies associated with mechanochemical reactions, such calculations are extremely computationally expensive, limited to small system size consisting of few atoms, and have no or limited dynamics.

Most of the drawbacks associated with first-principles or DFT calculations can be circumvented, albeit at the expense of some accuracy, using reactive molecular dynamics (MD) simulations^44^. Reactive MD simulations of a dimeric 9-anthracene carboxylic acid (Di-AC) mechanophore-embedded polymer showed that the critical strain required for mechanochemical activation was related to the elongation of a C–C bond^33^. Reactive MD simulations of the [4 + 2] Diels–Alder cycloadditions between anthracene and dienophiles showed that increasing compressive stress induced more distortion of the anthracene C–C–H angles before the reaction occured^16^. Reactive MD simulations have also been used to investigate molecular deformation in shear-driven reactions. Reactive MD simulations complementing ball-on-flat tribometer experiments captured significant distortion of C–C bonds lengths in allyl alcohol^45^ and α-pinene^29,46^ molecules prior to shear-driven oligomerization reactions. Despite this progress, a clear connection between molecular deformation and mechanical energy is missing, especially for shear-driven reactions where the in situ stress and energy states are complex.

Our previous research on cyclohexene explored shear-driven oligomerization reactions on stainless steel in inert, oxidizing, and reducing environments, and on hydroxylated silica in inert environment^47,48^. These studies showed easier mechanochemical activation during sliding for cyclohexene than for similar molecules such as cyclohexane and methylcyclopentane on both stainless steel and hydroxylated silica. Complementary reactive MD simulations revealed the shear-driven reaction pathways and identified the C=C double bond of the cyclohexene molecule as the source of its shear sensitivity^47^. However, the role of molecular deformation in mechanochemical activation within the context of the Bell model was not explored.

Therefore, this work aims to understand the effect of molecular deformation on the energetics of shear-driven mechanochemical reaction. Specifically, mechanochemical oligomerization of cyclohexene molecules is studied using reactive MD simulations, complemented by ball-on-flat tribometer experiments in a vapor phase lubrication (VPL) condition, to investigate the shear activation of chemical reactions. Experiments confirm polymerization reactions are driven by shear stress and provided information about the products and pressure-dependence of the reaction. The simulations show similar trends and are analyzed with specific focus on identifying and quantifying shear-induced deformation. Finally, nudged elastic band (NEB) calculations are used to calculate the energy barrier for oligomerization reactions with undeformed or deformed reactants and the trends are analyzed to identify a relationship between the mechanical energy term in the Bell model and molecular deformation.

## Methods

### Experimental methods

Cyclohexene vapor, carried by an N_2_ stream, was supplied into a ball-on-flat tribometer at a partial pressure of 30% relative to its saturated vapor pressure at room temperature. The substrate used was a Si (100) wafer (Wafer World, Inc., West Palm Beach, FL, USA) with a 5–7 nm thermally grown oxide layer on top. Moisture volume concentration was about 18 ppm in the N_2_ carrier gas. A borosilicate ball (Thermo Fisher) with a diameter of 3 mm was used as the countersurface. Before conducting the tribotest, both the substrate and ball were sequentially cleaned with rinsing with acetone, ethanol, and DI water in sequence, and then blown dry with nitrogen and exposure to UV/O_3_ to remove any organic residue. The sliding speed was set at 3.2 mm/s and the sliding length was 2.3 mm. At this speed, the average flash temperature rise was estimated to be less than 3 ℃^48^. Further details of the experimental setup were described in a previous study^48^. The normal load was varied from 50 to 200 g, corresponding to an average Hertzian contact pressure of 0.23–0.37 GPa. This corresponds to shear stresses between 0.06 GPa and 0.09 GPa, consistent with the range reported previously for ball-on-flat tribometer experiments with similar materials, load, and geometry^27,45^. The yield of products remaining on the surface after 600 reciprocating cycles of sliding was calculated by measuring the height above the reference plane using atomic force microscopy (AFM; Digital Instrument MultiMode scanning probe microscope; Tip model: TESPA-V2 Bruker)^32^. The yield was then normalized by the contact area and the total sliding time. Elemental analysis of the tribopolymer was performed using Energy Dispersive X-ray Spectroscopy (EDX) from Thermo Fisher Scientific (Model: Apreo S).

### Simulation methods

MD simulations of cyclohexene molecules sheared between two silica slabs were conducted using the Large Atomic/Molecular Massively Parallel Simulation (LAMMPS) software^49^. The timestep was 0.25 fs. The interactions between atoms in the simulations were modeled using the ReaxFF force field^50^ with a set of parameters previously developed^51^ from a combination of parameters for C/H/O^52,53^ and Si/C^54^ interactions. The forcefield used here^51^ was reparametrized based on the three source forcefields^52–54^ to model uniaxial tension and compression of solid polytetrafluoroethylene and liquid polydimethylsiloxane polymers. The reparameterization process involved direct comparison of ReaxFF model predictions to DFT calculations. For example, the C–C, C–H, and C–O bond compression/extension energies were compared with corresponding DFT calculations during the reparameterization process until satisfactory agreement was achieved^51^. This force field has been used to study mechanochemical reactions in similar systems, specifically the shear-driven oligomerization of α-pinene^29,46^, cyclohexane, methylcyclopentane, and cyclohexene^47,48^. Postprocessing of the simulation data was carried out using in-house python scripts and OVITO software^55^.

**Figure 1:**
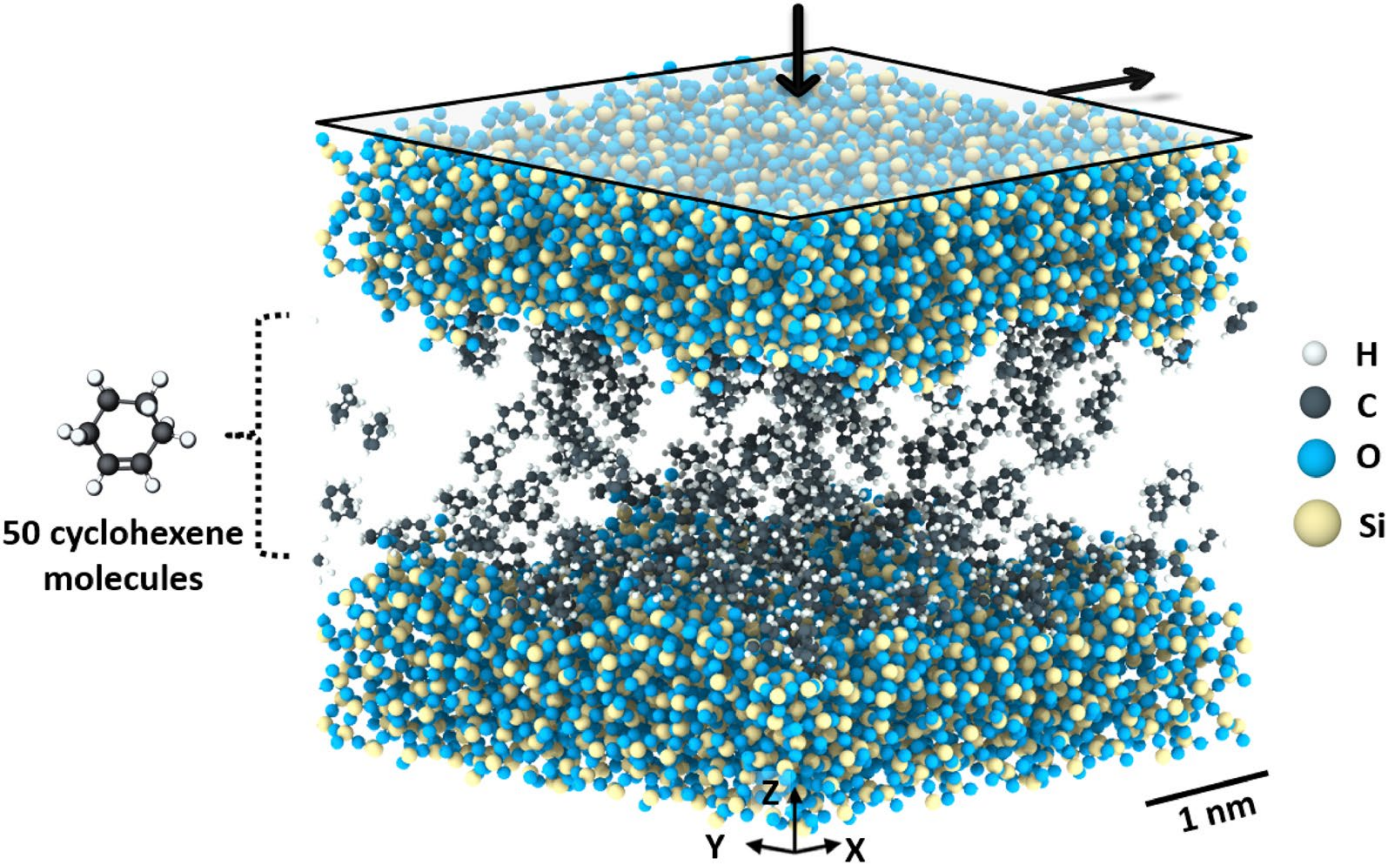
Simulation model consisting of two silica slabs and 50 cyclohexene molecules. The white, black, blue, and yellow spheres represent hydrogen, carbon, oxygen, and silicon atoms, respectively. The arrows show the direction of the applied normal and shear stress.

The model system, shown in Fig. 1, consisted of 50 cyclohexene molecules confined between two amorphous silica slabs. The number of cyclohexene molecules was chosen to form at least a monolayer coverage on the surfaces. The creation of the amorphous silica slabs involved using the heating and quenching method^29,46,47^. Bulk cristobalite (periodic boundaries in all three directions) was heated to 4000 K with a heating rate of 0.01 K/fs, followed by a 200 ps equilibration at 4000 K. Then the liquid was cooled down to room temperature in three steps: 4000 K to 1500 K at 0.005 K/fs rate, followed by a 250 ps equilibration at 1500 K, and finally, 1500 K to 300 K at 0.002 K/fs rate. A vacuum region was placed on two sides of the bulk cristobalite during the amorphization process to achieve a silica slab with an average roughness of ~ 2 Å. The slab was then equilibrated at 300 K and truncated from one side to have a thickness of ~ 20 Å. The slab was duplicated, and the two resulting silica surfaces were oriented as shown in Fig. 1.

The two silica slabs were initially placed 20 Å apart. This arrangement aimed to minimize interactions between the slabs, allow enough space for the cyclohexene molecules to equilibrate without mechanical stress, and minimize the computational time required to bring the slabs together. Silica atoms located at the bottommost 5 Å of the lower slab and the uppermost 5 Å of the upper slab were treated as rigid bodies. The initial dimensions of the simulation box were 33 × 32 × 71 Å^3^, with periodic boundary conditions in the x- and y-directions and fixed boundaries in the z-direction. The canonical ensemble (NVT) was applied to all non-rigid atoms, and charge equilibration was performed throughout the simulation.

A three-step procedure was followed for each MD simulation. First, energy minimization of the simulation system was performed using a conjugate gradient algorithm followed by dynamic equilibration at 300 K. Then, the upper slab was moved towards the bottom slab in the z-direction until the average distance between the slabs was approximately 10 Å. Next, the system was compressed at pressures of 1, 2, 3, or 4 GPa by applying load on top of the upper slab for 100 ps to let the system equilibrate at the desired pressure. Finally, sliding simulations were carried out by moving the upper slab at a speed of 10 ms^−1^ in the x-direction for 2 ns while keeping the normal load unchanged. Although the sliding speed used in the simulations is orders of magnitude higher than in the tribometer experiments, such sliding speed is routinely used in reactive MD simulations to minimize the computational cost. Simulations were repeated to get four independent trajectories at each pressure condition.

### Nudged elastic band methods

The energy barriers for oxidation and oligomerization reactions were computed using NEB calculations^56–58^. The NEB calculations were performed using LAMMPS with the same ReaxFF potential as in the dynamic simulations. The NEB method calculates the Minimum Energy Path (MEP) for any transition by optimizing intermediate images or replicas along the reaction path.

The NEB calculations were performed with a total of 50 replicas, including the initial and final replicas. The replicas were connected by virtual springs to ensure equal spacing between them. Another spring perpendicular to the transition path was applied to maintain a straight path for transition. The total force acting on a replica was the combination of the spring force along the tangent to the replica on the reaction path, the true force perpendicular to the tangent, and the perpendicular component of the spring force regulated by a switching function^57,59^. The series of replicas was converged to the MEP by minimizing the total force acting on each of the replicas through damped dynamics until a force criterion of 0.1 eV/Å was met for the saddle point^58–60^. The NEB calculations incorporated the climbing image method, where the replica with the highest energy was driven to the top of the energy barrier to maximize its energy.

Two sets of calculations were performed. In the first, the initial and final replicas for the NEB calculations were obtained directly from the reactive MD simulations. All silica atoms and cyclohexene molecules except for those directly relevant to the reaction were deleted from the simulation to create the initial and final replicas for the NEB calculations. The initial and the final replicas were energy minimized and structurally optimized in LAMMPS prior to the first set of NEB calculations. Then, a second set of calculations was performed for deformed cyclohexene. In this set, the structurally optimized cyclohexene molecule in the initial replica was systematically deformed by compressing or extending one or more specific bonds.

## Results and discussion

**Figure 2:**
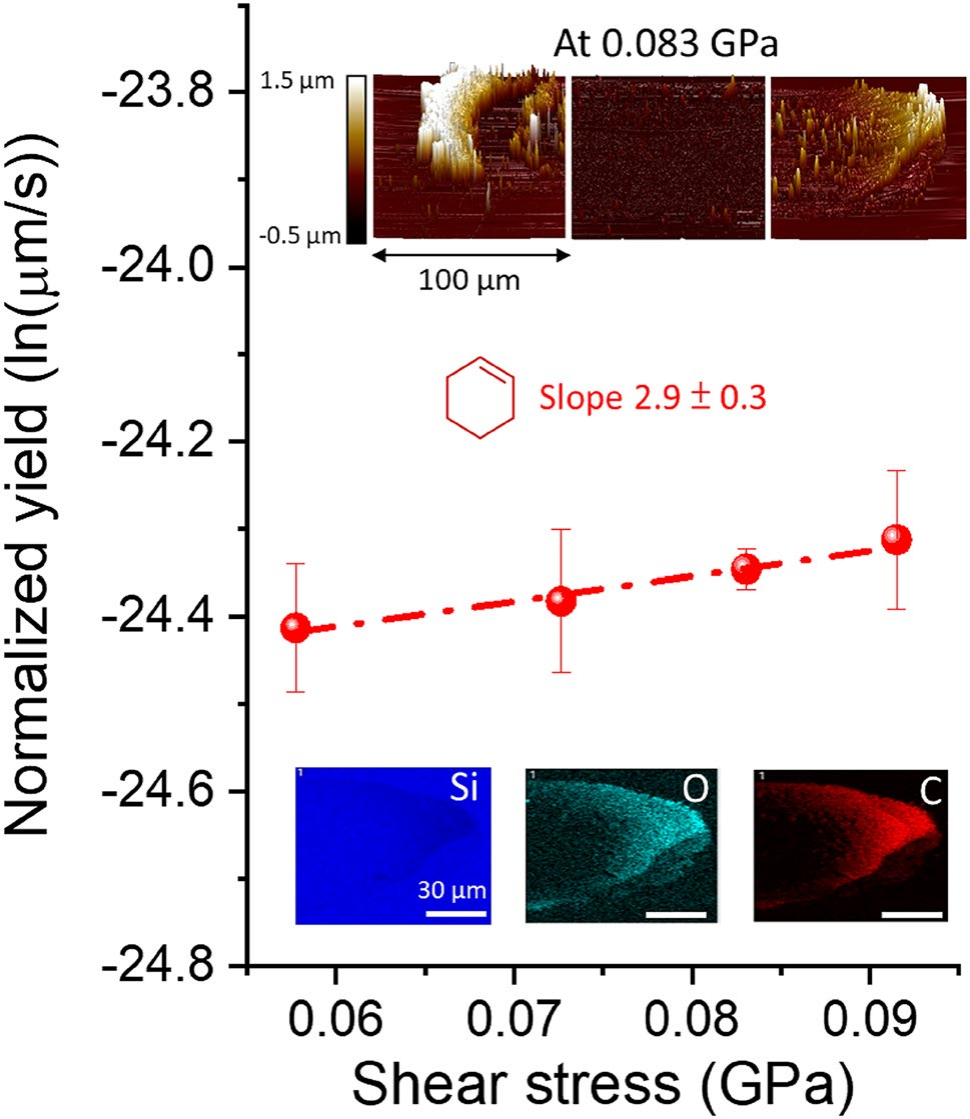
Semi-log plot of the normalized yield to shear stress for tribopolymers produced from cyclohexene in N_2_. Insets show AFM images of the left, middle, and right sides of the wear tracks, along with EDX mapping of the tribopolymer. Error bars represent the standard error of mean obtained from three different sliding tracks.

In the VPL experiment, products with low vapor pressure remain on the surface while species with high vapor pressure, such as unreacted cyclohexene adsorbates or small fragments, evaporate into ambient air during the tribotest. The yield of products that remain on the surface after the tribotest can be measured from the AFM tapping mode images (see inset to Fig. 2). Figure 2 shows the semi-log plot of normalized yield vs. shear stress from these experiments. The increase of yield with shear stress and the negligible temperature rise^48^ confirm this is a stress-activated reaction. Since only the mechanical energy term in Eq. (1) depends on shear stress, the slope of the line in Fig. 2 can be used to calculate the mechanical energy *E*_*m*_ to be between 0.1 kcal/mol and 0.2 kcal/mol for the range of shear stresses in the experiments.

EDX mapping revealed that the tribopolymer contains a significant amount of oxygen (see inset to Fig. 2), although the precursor (cyclohexene) does not have any oxygen. There are three possible origins. One is the involvement of surface oxygen in tribochemical reactions. The second is the oxidation of intermediates due to a trace amount of O_2_ and H_2_O in the nitrogen carrier gas. In fact, when the carrier gas was switched to O_2_, the reaction yield was observed to increase^48^. The third is post-test oxidation of chemically-unstable tribopolymers by ambient air during the sample transfer from the environment-controlled tribotesting unit to the EDX system. Although the current experiment cannot determine which is the main mechanism, previous reactive MD simulations suggested that the involvement of surface oxygen is likely to occur^29^. In fact, we have observed wear of the substrate when tribochemical polymerization of cyclohexene takes place under the sliding conditions, whereas no wear is observed in the absence of tribochemical polymerization^48,61^.

**Figure 3:**
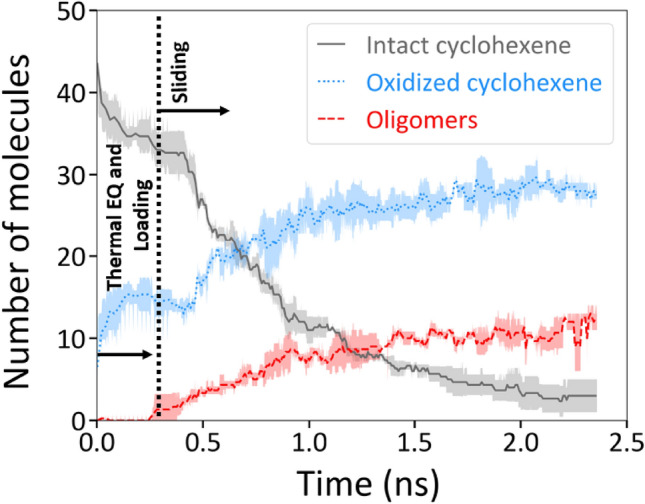
Evolution of intact cyclohexene molecules and reaction products in simulations at 300 K and 4 GPa normal stress. The number of intact cyclohexene molecules in the system decreased as the molecules underwent oxidative chemisorption or oligomerization reactions. Simulations were repeated four times at 4 GPa, and the lines represent the average of four simulations. The shaded regions represent one standard deviation from the average. The sliding process started at ~ 0.35 ns.

Atomistic details of the initial stages in the cyclohexene polymerization reactions were investigated with reactive MD simulations. These reactions were found to proceed in two steps^29,47^: oxidative chemisorption (corroborated by the EDX results) followed by oligomerization (specifically the formation of dimers, trimers, etc.) that are assumed to be the first step of tribopolymerization observed experimentally. Figure 3 illustrates the decrease in the intact cyclohexene molecules and the concurrent increase in the number of oxidized molecules and oligomers in simulations at 4 GPa normal stress. The number of oligomers was calculated based on the size of the product, e.g., dimers were counted as one, trimers as two, etc. The results of all simulations performed at 1–4 GPa normal stress conditions are shown in Fig. S1.

Approximately 10 cyclohexene molecules underwent oxidative chemisorption reactions even before any mechanical stress was applied during the initial thermal equilibration stage in the first 0.1 ns of a simulation (Fig. 3, S1b) due to the presence of reactive sites on the silica surfaces. After the initial 0.1 ns, normal stress was applied to the system and the cyclohexene molecules were compressed to the desired pressure. The application of normal stress resulted in a small increase in the chemical reactivity in the system. However, normal stress alone induced fewer than five oxidative chemisorption reactions (Fig. 3, S1b) and fewer than three oligomerization reactions (Fig. 3, S1c) in most simulation cases. A previous study on α-pinene investigated the effect of normal stress on similar oxidative chemisorption and oligomerization reactions and showed that, as seen here, normal stress alone was not a key driver of these reactions^29^.

In contrast, the application of shear stress at around 0.35 ns led to a notable increase in all reaction kinetics. With shear stress, the reduction of intact cyclohexene (Fig. 3, S1a) and the production of oligomers (Fig. 3, S1c) followed an exponential decay curve, indicating first-order kinetics. The temporal change of intact molecules in simulations was fitted with an exponential function to calculate the reaction rate constants for the consumption of intact reactants (Fig. S1a). Shear stress was averaged over the last 1.5 ns of each simulation when the friction in the system reached a steady state after an initial run-in stage (Fig. S2). Then, as was done with the yield of the polymerization reactions in experiments, we plotted the natural log of the reaction rate constant as a function of shear stress (Fig. S3). The reaction rate constant increased exponentially with increasing shear stress, which confirmed that the oxidative chemisorption and oligomerization reactions were stress-assisted. The mechanical energy *E*_*m*_ was calculated from the slope of a linear fit to the reaction rate constant vs. shear stress data (Fig. S3) and was found to be between 0.4 kcal/mol and 1.8 kcal/mol with shear stresses ranging from 0.7 GPa to 2.2 GPa.

The mechanical energy contribution lowering the effective activation energy is larger in the simulation than the value determined in the experiments. However, considering the large difference in sliding speed, system scale, and the number of reactants, the observed discrepancy is relatively minor. In addition to scale, there are several other factors that could contribute to the difference in mechanical energy. First, the experimental values were calculated using the average contact stress estimated from the Hertzian contact mechanics because the distribution of local contact stress due to the surface roughness could not be determined. Second, once tribopolymers were produced in experiments, not all of them could be pushed out to the ends of the sliding track (see Fig. 1) and some would remain in the track. The latter could affect the adsorption isotherm of cyclohexene to the surface and be involved in subsequent reactions. Lastly, the mechanical energy calculated in the simulations was for the “consumption” of monomers, but that obtained in the experiment was for the “production” of polymeric species. Not all cyclohexene molecules activated by oxidative chemisorption end up forming oligomers; some of them must have desorbed back into the gas phase during the interval between consecutive sliding cycles. Despite the difference in the absolute values of *E*_*m*_, a similar trend in reaction yield or rate constant with respect to shear stress suggests that atomistic details found by tracking atomic trajectories in MD simulations are relevant to dynamic processes occurring in the experiment^45,47^.

The shear stress dependance of the cyclohexene oligomerization is consistent with the previous studies where such mechanochemical oligomerization or polymerization of organic molecules at sliding interfaces has been shown to be primarily shear driven^44,48,62^. Previous reactive MD simulations have shown that oligomerization of allyl alcohol and α-pinene were driven by shear stress^29,45,46^. The effect of normal and shear stress was further decoupled in case of α-pinene using reactive MD simulations by applying cyclic normal load which showed that normal stress alone could not drive chemical reactions^29^. Shear sensitivity has also been observed for commercially used organic molecules with complex chemical structure. Polymerization of zinc dialkyl dithiophosphate or tricresyl phosphate on various metallic and non-metallic surfaces during rubbing motion has been shown to follow the Bell model where the primary stress component to drive the reactions was shear stress^26,27,63–65^. While it is evident that shear stress can effectively activate organic molecules to undergo oligomerization reactions, the mechanism through which shear activation happens requires further investigation.

**Figure 4:**
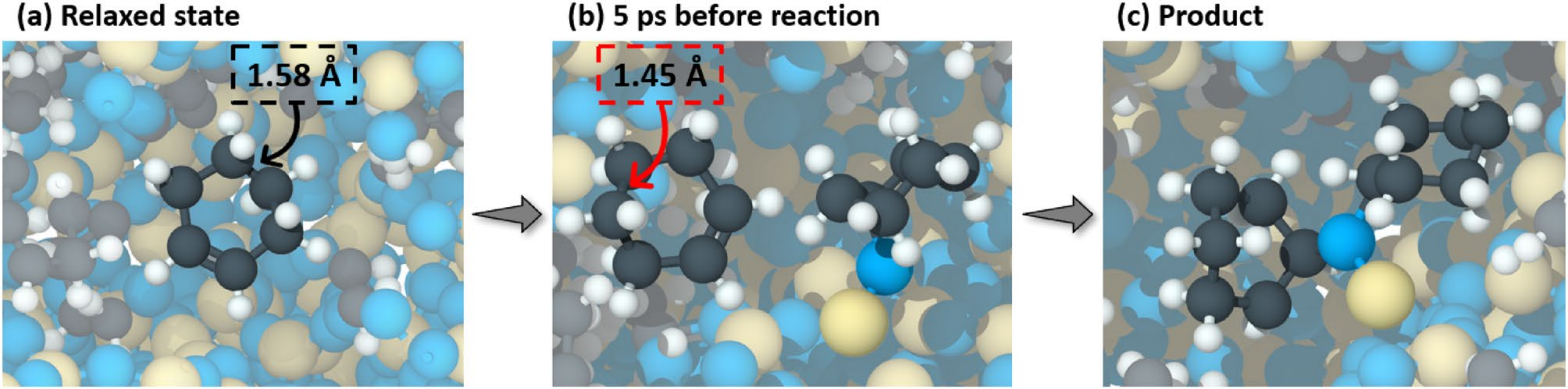
Simulation snapshots of an intact cyclohexene molecule showing a series of events that led to an oligomerization reaction. (**a)** The intact molecule in relaxed state where all bond lengths and angles were close to their equilibrium values. The length of one of the C–C bonds at the relaxed state was ~ 1.58 Å. (**b)** The intact molecules within 5 ps prior to oligomerization. The previously mentioned C–C bond was deformed by shear and its length decreased to ~ 1.45 Å just before the reaction happened. (**c)** The deformed molecule reacted at the C=C double bond with an oxidized molecule and formed a dimer.

To understand the mechanisms of shear-activation, reactions between intact cyclohexene molecules and chemisorbed cyclohexene molecules were monitored to determine the reaction pathway. Snapshots of a representative reaction are depicted in Fig. 4 and the corresponding chemical equation for the reaction is provided in Fig. S4. Previous reactive MD simulations have shown that shear-driven oligomerization reactions of cyclohexene on silica involve direct activation of the C=C bond^47^. Similar reaction pathways were observed in this study where the carbon atoms of the double bond reacted with a chemisorbed molecule (Fig. 4b) to create an ether linkage and consequently produce an oligomer (Fig. 4c). The molecular structure of the intact cyclohexene molecule participating in oligomerization reactions was closely analyzed throughout the reaction process to detect deformation. Notably, within 5 ps of product formation, one of the C–C bonds opposite to the C=C bond in the cyclohexene became visibly distorted compared to the thermally relaxed molecule (Fig. 4b).

**Figure 5:**
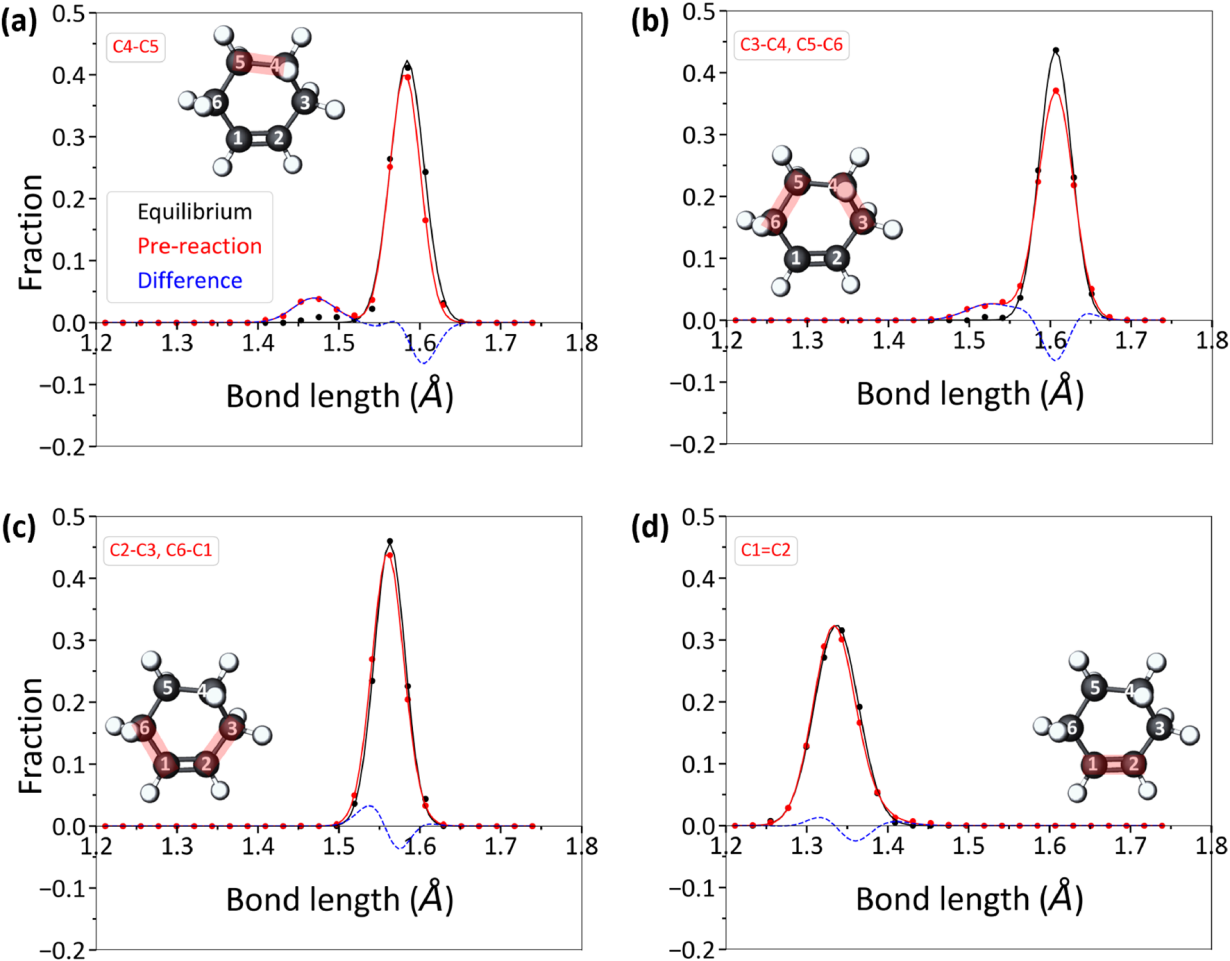
Bond length distributions of cyclohexene molecules at equilibrium (black distributions) and prior to oligomerization reactions (red distributions). The equilibrium distributions were calculated from intact cyclohexene molecules in the first 50 ps of simulation during which no mechanical stress was applied. The red distributions were calculated from intact cyclohexene molecules that were within 5 ps of participating in an oligomerization reaction. The blue dotted lines show the difference between the red (pre-reaction) and black (equilibrium) distributions.

To substantiate the findings, the distribution of bond lengths in the cyclohexene molecules within 5 ps of an oligomerization reaction were compared with the bond length distributions for the cyclohexene molecules at thermal equilibrium (Fig. 5). This analysis has been previously used to detect molecular deformation of allyl alcohol and α-pinene^45,46^. Here, the carbon atoms in cyclohexene were numbered from 1 to 6 according to the IUPAC convention, where C1 and C2 correspond to the carbon atoms at the double bond site. Since the cyclohexene molecule is symmetrical, data for C2-C3 and C6-C1 bonds, and C3-C4 and C5-C6 bonds were combined to create one distribution for each pair of bonds (see insets to Fig. 5).

In Fig. 5, the blue dashed lines highlight the difference between the equilibrium and pre-reaction bond length distributions, where a positive value means there were more bonds with a given length just before the reaction than at equilibrium. The blue dashed line for the C4-C5 bond shows an increased population at 1.46 Å, which is shorter than the average equilibrium bond length of 1.58 Å. This indicates this bond was compressed just before reaction (Fig. 5a). Note that the ReaxFF forcefield utilized in this study slightly overestimated the average equilibrium bond length of the C–C single bonds in cyclohexene^66^. The distribution for C3-C4 and C5-C6 bond lengths exhibited a shoulder peak around 1.52 Å (Fig. 5b), indicating these bonds too were compressed slightly before the reaction. The C2-C3 and C6-C1 bonds, adjacent to the double bond, did not exhibit an appreciable change in length (Fig. 5c). Similar trends in bond length distributions were observed in the bond-by-bond analysis for oxidative chemisorption (Fig. S5). Note that this analysis was only carried out for the intact cyclohexene molecules since the chemisorbed molecules already exhibited non-equilibrium structures so there was no reference state.

Although the C1=C2 double bond site was the most reactive under shear, the C1=C2 bond length distribution prior to reactions was similar to the equilibrium distribution (Fig. 5d) and did not show any signs of deformation. However, physical deformation leading to mechanochemical activation may or may not involve the stress-activated bond. For example, the physical deformation required for the mechanochemical decomposition of methyl thiolate occurred at the angle between the surface and the scissile S-C bond^14^. Mechanochemical activation of spiropyran was shown to involve the deformation of dihedrals in the vicinity of but not directly at the scissile C-O bond^67^. A similar effect has been observed for polymers where the accumulation of strain in dihedrals facilitated bond dissociation^68^. Further, the shear-activation of the C=C double bond of α-pinene has been shown to be triggered by deformation of C–C bonds or dihedrals at the four membered or six membered ring^29,46^. So, the role of deformation in mechanochemical activation may not be to directly weaken the reacting bond, but rather to alter the potential energy surface of the reactant molecule such that a specific bond or chemical feature can be easily activated.

**Figure 6:**
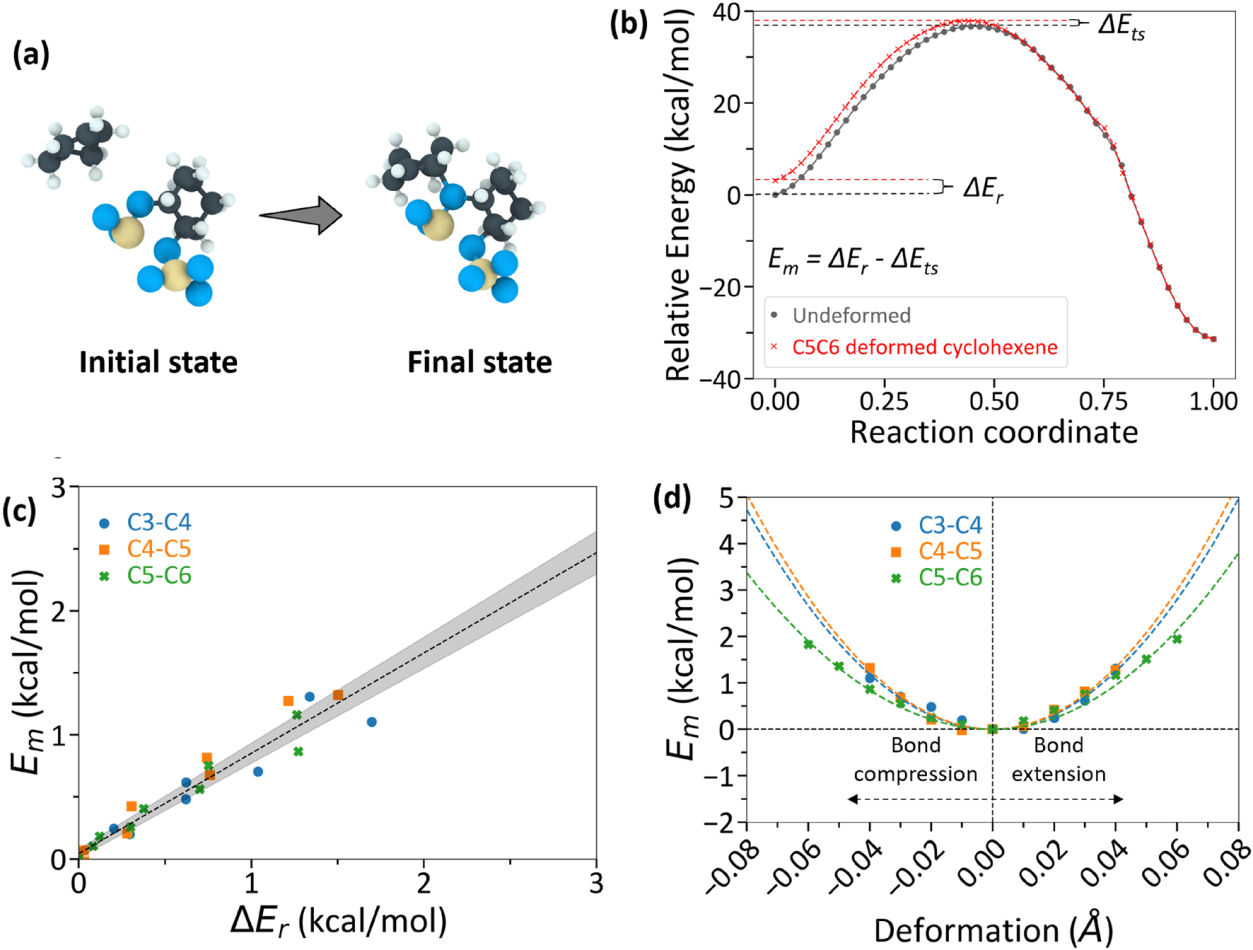
NEB calculation results for a representative oligomerization reaction. (**a)** The initial and final states of an oligomerization reaction used for NEB calculations, (**b)** Representative NEB-calculated MEP for structurally optimized, undeformed cyclohexene molecule (black) and for deformed cyclohexene molecule (red). For the deformed case, the C5-C6 bond of the intact cyclohexene molecule was compressed by ~ 0.06 Å from the average equilibrium bond length. Variables are defined in the text. (**c)** Mechanical energy, E_m_, vs. change in reactant state energy, ΔE_r_, from NEB calculations. Each data point represents an individual NEB calculation with compressive deformation to a specific bond. The fitted dashed line had an R^2^ value of 0.93 and a slope of ~ 0.81, suggesting that E_m_ ≈ ΔE_r_. (**d)** NEB calculated reduction in energy barrier, E_m_, for a range of deformed bond cases. E_m_ increased quadratically (dashed lines show quadratic fit) with increasing bond deformation.

To quantify how much the reaction energy barrier was reduced by molecular deformation, NEB calculations were conducted for oligomerization reactions using undeformed and deformed cyclohexene molecules. The NEB calculations were performed with the ReaxFF potential such that the results could be directly correlated to observations from the ReaxFF MD simulations. The initial reactant state and the final product state of the NEB calculations are shown in Fig. 6a and representative NEB-calculated MEPs for undeformed and deformed cyclohexene molecules are shown in Fig. 6b. Since deformation was mostly observed in the C3-C4-C5-C6 side of a reactant cyclohexene molecule in Fig. 5 and S5, the NEB calculations were performed with the C3-C4, C4-C5, or C5-C6 bond deformed by ± 0.01 Å up to a maximum of ± 0.06 Å.

The mechanical energy (*E*_*m*_) determined from the slope in the semi-log plot of reaction rate constant vs. the shear stress is the difference between the change in reactant energy $\Delta$ *E*_*r*_ and the change in transition state energy $\Delta$ *E*_*ts*_ due to mechanical stress, which can be expressed as:

2 $${E}_{m}=\Delta {E}_{r}-{\Delta E}_{ts}$$

An increase in reactant energy or a decrease in transition state energy could cause an increase in the total mechanical energy. For the representative NEB calculation with deformation shown in Fig. 6b, where the C5–C6 bond was compressed by 0.06 Å, the reactant energy increased by $\Delta$ *E*_*r*_ = 3.1 kcal/mol and the transition state energy increased by $\Delta {E}_{ts}$ = 1.2 kcal/mol, such that the total mechanical energy was 1.9 kcal/mol. This is the amount by which the thermal activation energy was decreased by shear-induced deformation of the molecule. To determine if this trend was generalizable, we calculated ${E}_{m}$ and $\Delta$ *E*_*r*_ for all unique bonds in the cyclohexene molecule deformed from compression to tension (Fig. 6c). The plot exhibits a linear relationship with a slope of ~ 0.81 (R^2^ = 0.93) between *E*_*m*_ and $\Delta$ *E*_*r*_. The strongly positive correlation confirms that most of the mechanical energy is attributed to an increase in a reactant state energy. The slope being slightly below unity shows that deformation also increases the transition state energy, albeit much less than the amount by which the reactant energy increases. The findings here from ReaxFF-based NEB calculations are consistent with DFT calculations since an increase in the reactant energy and a smaller increase in the transition state energy due to reactant deformation has also been captured in DFT calculations of [4 + 2] Diels–Alder cycloadditions between anthracene and dienophiles under normal stress^16^.

Figure 6d illustrates the relationship between the mechanical energy from an NEB-calculated MEP and the amount of deformation introduced to individual C–C bonds in the reactant molecule. The mechanical energy varied quadratically with the amount of deformation in each C–C bond. As the deformation applied to the C–C bonds in the NEB calculations was small (< 0.1 Å) and no bonds dissociated in the deformed reactant molecule, the reactant molecule was modeled as a Hookean spring. Then, the mechanical energy could be considered as the energy stored in a spring subject to axial deformation. Such modeling approach using Hooke’s law has previously been used to explain the deformation behavior of mechanophores under applied stress^69,70^. Single molecule force spectroscopy of polymer chains using AFM has shown that, at small deformation (~ less than 0.3 strain), the stretching of a polymer chain could be modeled using Hooke’s law^69,70^. Consequently, the data in Fig. 6d were fitted with the quadratic potential energy equation of a spring as ${E}_{m}=K\Delta {x}^{2}$, where $\Delta x$ is the amount by which a bond is deformed from equilibrium and *K* is the force constant.

The average force constant obtained from the fitted lines in Fig. 6d was 9.8 mdyn/Å. Comparatively, the typical force constant for a C–C single bond is about 4 mdyn/Å while, for C=C, it is about 8.8 mdyn/Å^71,72^. Our calculated force constant was higher than the force constant reported for single bonds. However, the deformation of a C–C bond in the reactant cyclohexene molecule resulted in deformation of the adjacent bonds as well as bond angles in the NEB calculation, so the stored energy was associated with deformation of the entire molecule with a force constant larger than that of individual bonds. Regardless, the close agreement between the calculated and expected force constants suggests that the contribution of shear stress to mechanical energy can be quantitatively correlated to physical deformation of reactant molecules.

## Conclusion

In conclusion, studying the shear stress-driven reactions of cyclohexene on silica using reactive MD simulations and ball-on-flat tribometer experiments showed that cyclohexene molecules underwent oligomerization or polymerization reactions that followed the typical stress assisted thermal activation model. Reactive MD simulations highlighted the role of oxidative chemisorption in the reaction pathway, an observation that was corroborated by the presence of oxygen in the EDX mapping of the polymer products in experiments.

Results from the reactive MD simulations pointed to stress-induced molecular deformation as the mechanism of mechanochemical activation. Qualitative evidence of deformation was found by visually analyzing intact cyclohexene molecules prior to oligomerization. Deformation was then quantified by comparing all C–C bonds of the reactant cyclohexene molecules to the corresponding C–C bonds of a cyclohexene molecule in thermal equilibrium. Both visual and quantitative analysis captured physical deformation on the opposite side of the C = C double bond of the six-carbon ring of a reactant cyclohexene molecule prior to shear-driven oligomerization reactions.

The relationship between physical deformation of reactant molecule and mechanical energy in the Bell model was then investigated by a series of NEB calculations of an oligomerization reaction. Comparing the NEB-calculated MEP for all structurally optimized reactants with MEPs for systematically deformed reactant cyclohexene molecule showed that the reactant state energy increased considerably with deformation while transition state energy remained unchanged or increased only slightly relative to the reactant energy change. Then the NEB-calculated mechanical energy was quantitatively correlated to molecular deformation by modeling the mechanical energy as energy stored in a harmonic spring representing a cyclohexene molecule.

More generally, this study demonstrates the utility of experimentally corroborated reactive MD simulations to capture the dynamic aspects of shear-driven chemical reactions. Since these simulations are computationally efficient enough to model many reactant species sheared by non-ideal surfaces, they provide a means of statistically analyzing reaction pathways and molecular deformation, and a similar approach to that demonstrated here can be used to explore the fundamental mechanisms of other mechanochemical systems.

### Supplementary Information

Supplementary Information.

## Data Availability

Raw data from simulations and experiments can be provided upon reasonable request submitted to the corresponding author.
