# Supplementary material for: Shear-activation of mechanochemical reactions through molecular deformation
Source: Sci Rep. 2024 Feb 5;14:2992. doi: 10.1038/s41598-024-53254-2 (PMC10844542; doi:10.1038/s41598-024-53254-2)
Supplement: Supplementary file 1 — Supplementary Information. [file 41598_2024_53254_MOESM1_ESM.docx]

**Shear-Activation of Mechanochemical Reactions Through Molecular Deformation**

*Fakhrul H. Bhuiyan^1^, Yu-Sheng Li^2^, Seong H. Kim^2^, Ashlie Martini^1^*

*^1^Department of Mechanical Engineering, University of California Merced, 5200 N. Lake Road, Merced, California 95343, United States*

*^2^Department of Chemical Engineering and Materials Research Institute, Pennsylvania State University, University Park, Pennsylvania 16802, United States*

**Reaction kinetics**


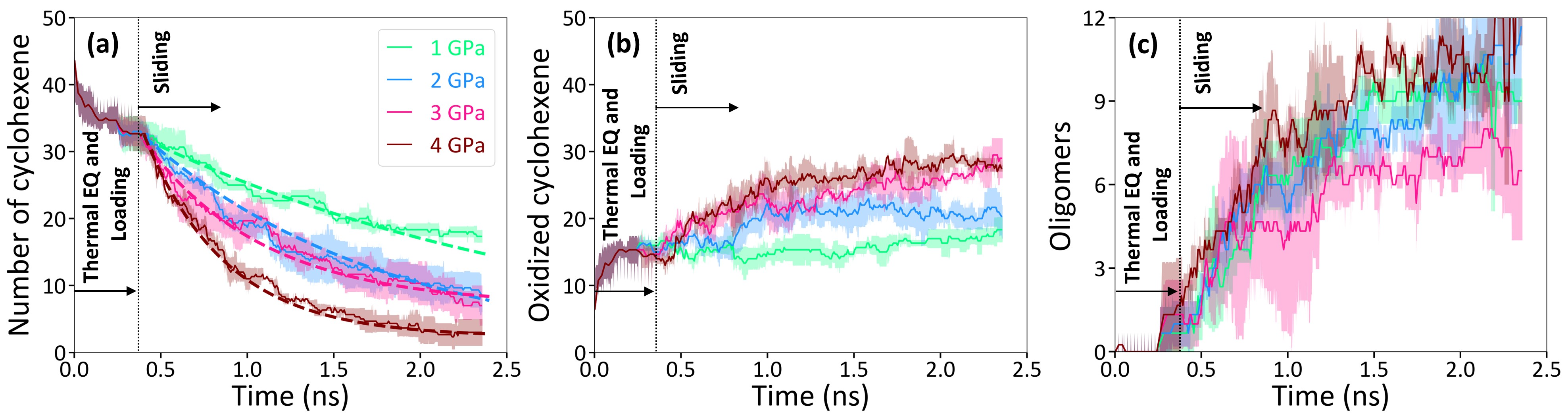


Figure S1: Reaction kinetics analysis from simulations at 300 K and four different normal stress conditions. (a) Decrease in number of intact cyclohexene molecules with simulation time. Evolution of (b) oxidized cyclohexene molecules and (c) oligomers in the simulations. Simulations were repeated four times for each normal stress condition and the solid lines in (a), (b) and (c) represent the average of four simulations. The shaded regions in (a), (b), and (c) represent one standard deviation from the average. The thicker dashed lines in (a) represent fits to the first order kinetics equation.


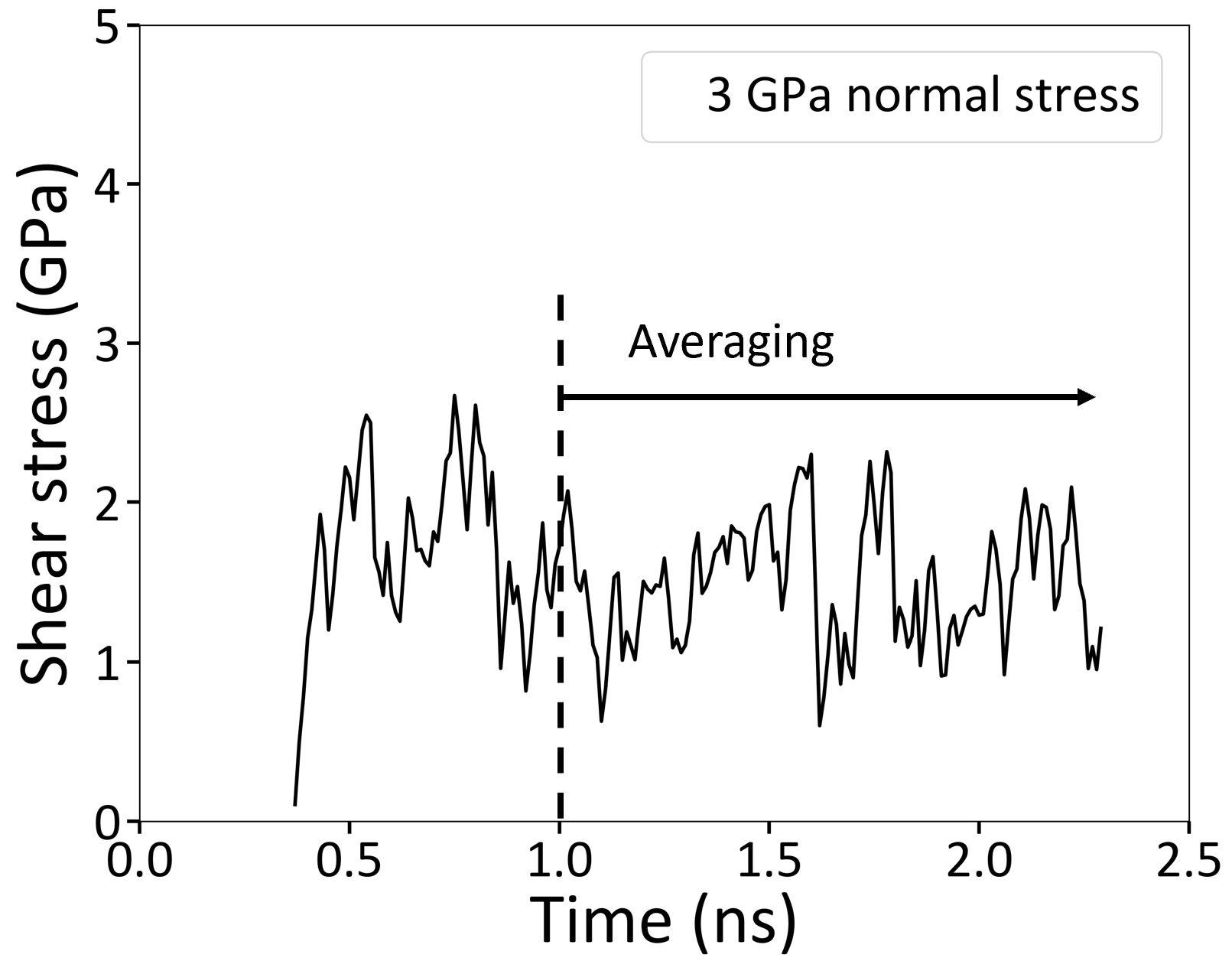


Figure S2: Representative plot of the shear stress in a sliding simulation at the 3 GPa normal stress condition. The average shear stress was calculated from the data after 1.0 ns of sliding.

**Mechanical energy, *E_m_*, and activation volume, *ΔV*,* calculations**

The rate of mechanochemical reaction can be expressed as an Arrhenius-type function, commonly known as the Bell model^1^:

$$k=A exp\left( -\frac{E_{a}-E_{m}}{k_{B}T} \right) (S1)$$

where, *k* is reaction rate constant, *A* is the pre-exponential factor, *E_a_* is activation energy, *E_m_* is the mechanical energy, *T* is temperature, and *k_B_* is the Boltzmann’s constant. *E_m_* can also be expressed as:

$$E_{m}=\tau\Delta V^{*} (S2)$$

where 𝜏 is shear stress and $\Delta V^{*}$ is the activation volume. Combining Equations S1 and S2 and taking natural log on both sides gives:

$$\ln(k)=\tau\frac{{\Delta V}^{*}}{k_{B}T}+\left( ln(A)-\frac{E_{a}}{k_{B}T} \right) (S3)$$

Therefore, on a semi-log plot of the reaction rate constant against the shear stress, the slope can be used to estimate *ΔV*^*^.^2^ The reaction rate constant, *k*, can be calculated by fitting the temporal change of the number of unreacted precursor molecules in the simulation to the first order kinetics equation, as shown in Fig. S1a. Shear stress, 𝜏, in the simulation can be calculated from the shear stress on the upper slab, as shown in Fig. S2.


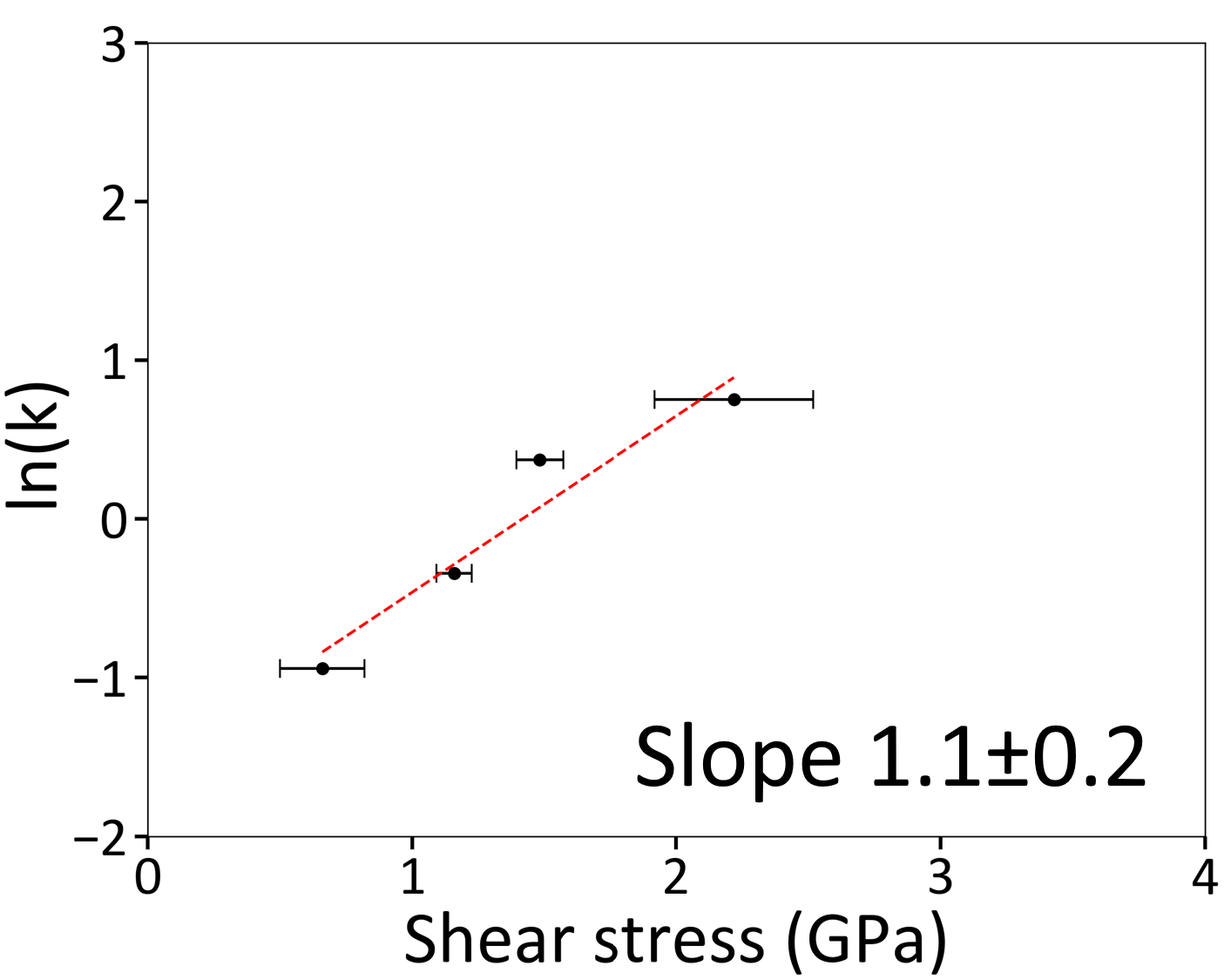


Figure S3: Activation volume for cyclohexene consumption calculated from simulations. The rate constants were calculated from Fig. S1a, and the shear stresses were calculated from sliding simulations at 1-4 GPa normal stress conditions, as shown by the representative plot in Fig. S2. The red dashed line in shows the fit to the Bell model (Equation S3).

**Chemical reaction formula**


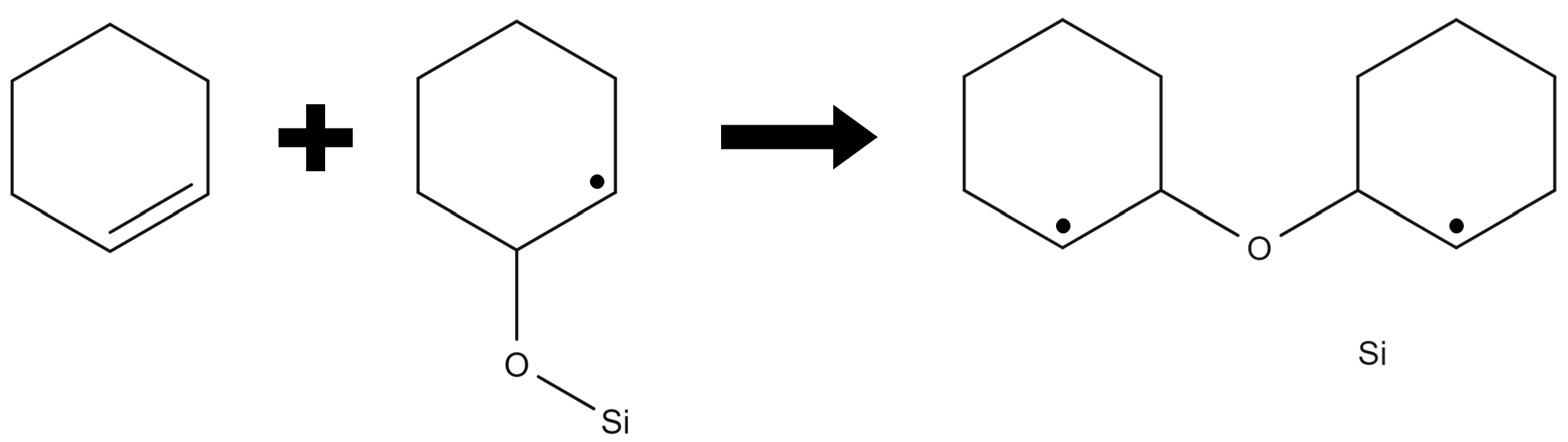


Figure S4: Reaction formula of the chemical reaction shown as simulation snapshots in Fig. 4; an intact cyclohexene molecule reacting with an oxidized, surface-chemisorbed molecule to form an oligomer.

**Bond-by-bond analysis for oxidation**


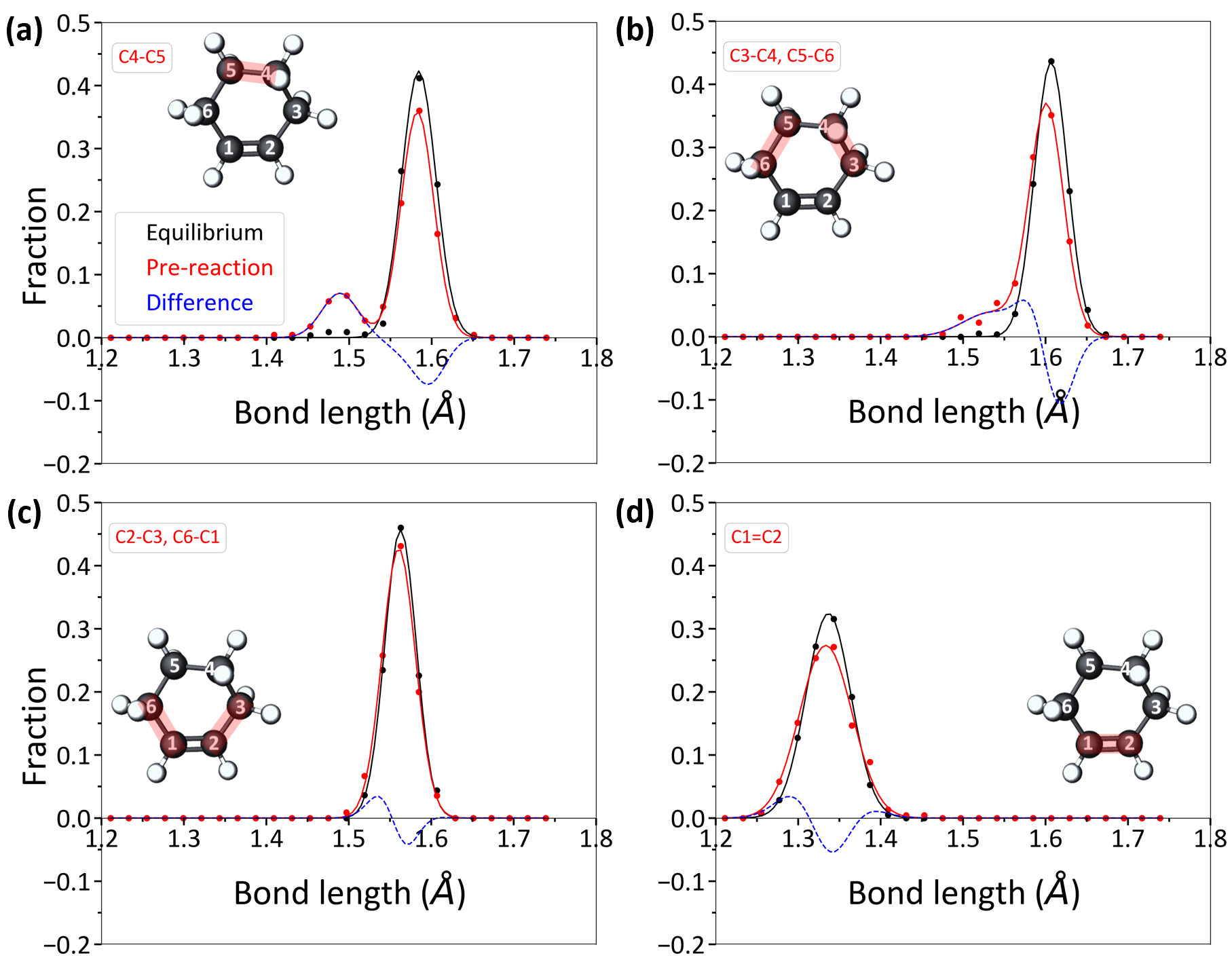


Figure S5: Bond length distributions of cyclohexene molecules at equilibrium (black distributions) and prior to oxidation reactions (red distributions). The equilibrium distributions were calculated from intact cyclohexene molecules in the first 50 ps of simulation during which no mechanical stress was applied. The red distributions were calculated from intact cyclohexene molecules that were within 5 ps of participating in an oligomerization reaction. The blue dotted lines show the difference between the red (pre-reaction) and black (equilibrium) distributions.

**References**

1 G. I. Bell, *Science*, 1978, **200**, 618–627.

2 A. Martini and S. H. Kim, *Tribol. Lett.*, 2021, **69**, 150.
